# Effect of Thermal Processing and Maceration on the Antioxidant Activity of White Beans

**DOI:** 10.1371/journal.pone.0099325

**Published:** 2014-07-03

**Authors:** Karina Huber, Priscila Brigide, Eloá Bolis Bretas, Solange Guidolin Canniatti-Brazaca

**Affiliations:** Agri-Food Industry, Food and Nutrition Department, Luiz de Queiroz College of Agriculture, University of São Paulo, Piracicaba, São Paulo, Brazil; Cairo University, Egypt

## Abstract

Phenolic compounds, which naturally occur in beans, are known to have antioxidant activity, which may be partially lost during the processing of this legume. This study evaluated the effect of thermal processing and maceration on the phenolic acid and flavonoids profile and content and on the antioxidant activity of white beans. According to the results obtained from the 2,2-azino-bis (3-ethylbenzothiazoline-6-sulfonic acid) diammonium salt (ABTS) method, there were no significant differences among treatment groups analysed. When was using 1,1-diphenyl-2-pycrylhydrazyl method (DPPH), beans cooked without maceration present the higher antioxidant activity, and raw beans the lower. The phenolic acids found in greater amounts were gallic acid and chlorogenic acid. Kaempferol was only detected in the soaked and cooked samples; catechin and kaempferol-3-rutinoside were found in the highest concentrations. Quercetin and kaempferol-3-glucoside were not affected by the cooking process, either with or without maceration. In general, the heat treatment increased the antioxidant activity.

## Introduction

Free radical formation, in the body, is controlled naturally by antioxidants. An antioxidant is substance that is able to regenerate an oxidized substrate. This effect is obtained with a low concentration compared with the substrate [1]. Antioxidants are able to stabilizing, or deactivating, free radicals before they interact with cells.

The organism has several mechanisms of antioxidant defense to control the excess of reactive oxygen species. Enzymes are one of them. The antioxidant enzymes are catalase, superoxide dismutase, glutathione reductase and glutathione peroxidise. For their activities some trace minerals are cofactors such as selenium, iron, copper, zinc and manganese. Food provides others substances like, alpha-tocopherol, ascorbic acid (vitamin C), beta-carotene and others carotenes and phenolic compounds [2]. The last one is the more importance in beans.

The predominant phenolic compounds in legumes are the flavonoids, phenolic acids and procyanidins [3]. Some polyphenols are specific to certain foods (e.g., flavanones in citrus fruits, isoflavones in soy), while others are found in various food matrices. A common bean contains phenolic acids such as p-coumaric, vanillic, caffeic, ferulic and sinapic acids, and flavonoids such as quercetin and kaempferol [3], [4]. The amount and composition of flavonoid glycosides, condensed tannins (procyanidins) and anthocyanidins determine colour of the tegument [5]. Dark-coloured beans have higher levels of phenolic compounds than the light-coloured ones [6]. In general, foods contain a complex mixture of phenolic compounds, which limit the assessment of the foods' bioavailability and biological activity due to the presence of synergistic compounds [7].

The processing condition commonly applied to beans not only improves their sensory aspects but also inactivates trypsin inhibitors and hemagglutinin [8]. Furthermore, this processing condition leads to changes in both the physical characteristics and the chemical composition of the beans. Beans are macerated before cooking, thus reducing cooking time results in beans that are softer in texture. Pressure cooking can improve the quality of beans by improving the taste and colour, inactivating antinutritional components while activating other biologically active compounds [9]. Although maceration prior to cooking reduces the levels of phenolic compounds naturally present in raw beans, it also leads to a reduced content of antioxidants [6]. Approximately 61% of flavonoid in raw beans were destroyed during cooking, 30 to 40% of polyphenol are lost in cooking water, and 75 to 79% of these compounds are leached into soaking water, which is then discarded [9].

This study evaluated the effect of thermal processing and maceration on the phenolic profile and content, and antioxidant activity of white beans.

## Material and Methods

### Study design

A common bean (*Phaseolus vulgaris* L.), strain G-2358 (white), was separated into six portions, four were cooked and two were raw. The beans were analysed raw and thermally processed (cooked).

The beans used for the analyses were divided into three treatment groups, each group containing two portions: treatment 1 raw beans was used; treatment 2 consisted of 10-hour maceration of beans in distilled water, with subsequent water replacement and cooking in an autoclave at 121°C for 10 minutes and treatment 3 consisted of cooking beans in an autoclave at 121°C for 10 minutes. After cooking, the samples were lyophilised and stored at 4°C. The cooked and raw beans were ground in a knife grinder and sieved through 30 mesh until they became bran, in the same conditions before analysis.

### Extract preparation

The extracts were obtained according to the method by Cardador-Martinez et al. [10]. Per 10 grams of ground sample, 100 mL of methanol was added and stirred at 70 rpm for 24 hours at 25°C, and the procedure was done twice for each portion. The samples were centrifuged for 10 minutes at 812x*g* before supernatant was evaporated in a rotary evaporator (Fisatom São Paulo Brazil). The extracts were lyophilised and subsequently analysed.

### Separation of fractions from extract

Half a gram of the lyophilised extract was diluted in 1 mL of methanol and then poured into the open column containing silica gel (Sigma Aldrich, 13% CaSO_4_). An eluting solvent (100 mL) with increasing polarity from petroleum ether, ethyl acetate, a mixture of ethyl acetate/methanol in various ratios to methanol was used to obtain six fractions (identified as A, B, C, D, E and F) according to a methodology reported by Aparicio-Fernandez et al. [11]. Fraction A was discarded because it contained a fraction of lipids used for cleaning the column. Fractions D and E do not present a high antioxidant activity. Each fraction was concentrated in a rotary evaporator for elimination of solvent and the resulting fractions were then lyophilised.

### Determination of antioxidant capacity using radical DPPH

The antioxidant activity was determined using the 1,1-diphenyl-2-pycrylhydrazyl (DPPH) method, as reported by Brand-Williams et al. [12]. The results were expressed as milligrams of Trolox equivalents antioxidant capacity (TEAC) g^−1^ extract.

### Determination of antioxidant capacity using radical ABTS

We also determined the antioxidant activity using the 2,2-azino-bis (3-ethylbenzothiazoline-6-sulfonic acid) diammonium salt (ABTS) that was reported by Re et al. [13], Van Den Berg et al. [14] and Arts et al. [15] with some modifications. The cation ABTS^+^ was prepared by mixing 6.62 mg of potassium persulfate (2.45 mM) and 38.4 mg of ABTS (7 mM) in 10 mL of deionized water, and subsequently kept in the dark for 12 to 16 hours at 25°C for release of the ABTS radicals. The Trolox standard solution was prepared by dissolving 5 mg of Trolox in 10 mL of ethanol. To preparing the samples 1.0 g sample was weighed and diluted in 10 mL of ethanol, shaken in shaking platform for 15 minutes and then transferred to centrifuge tubes, and centrifuged for 10 minutes at 519×g, supernatant was used. Prior to the readings of the samples, the ABTS^+^ was diluted in ethanol at a ratio of 1∶99, and the absorbance adjusted to 0.700±0.002 in the spectrophotometer at 734 nm. Twenty mL of supernatant of the each sample was added 2 mL of dilute ABTS solution. Reading at 734 nm for 5 minutes and zero time was taken. A standard curve was constructed using concentrations of Trolox 250–2000 mM, ethanol and ABTS. The results were expressed as milligrams of Trolox equivalents antioxidant capacity (TEAC) g^−1^ extract.

### Identification and quantification of phenolic acids

For the identification and quantification of phenolic acids, we separated the free phenolic acids using High Performance Liquid Chromatography (HPLC), according to a method reported by Xu and Chang [16] with some modifications. The chromatograph used was a Shimadzu model 20A that was equipped with a UV detector (270 and 325 nm). For the separation, we used an analytical Column Zorbax ODS Stablebond-C18 (Agilent Technology) (4.6 mm×250 mm) at 40°C. The mobile phase consisted of A (0.1% trifluoroacetic acid solution in water) and B (100% methanol). An isocratic gradient was used (20% to 80% of A and B) with a flow rate of 1.00 mL minute^−1^.

To identify the samples peaks on HPLC, a stock solution (1 mg mL^−1^) of each phenolic acid was individually prepared and diluted. The diluted solutions were injected (20 µL) separately and in duplicate, as described above. The resulting peak areas and their retention times were used for the comparison, identification and quantification of phenolic compounds in the extracts.

### Preparation of stock solutions for phenolic acids

To preparing the stock solution, 10 mg of each phenolic acid was dissolved in 10 mL of 80% methanol and then serially diluted in 80% methanol: 0.5, 1, 2.5, 5, 10 and 25 mg of vanillic acid mL^−1^; 10, 25, 50 and 100 µg mL^−1^ of chlorogenic acid; 1, 2.5, 5, 10, 25, 50 and 100 µg mL^−1^ of sinapic acid; and 1, 5, 10, 25 and 50 µg mL^−1^ of gallic acid. The content of phenolic acids was expressed as micrograms per gram of extract (µg g^−1^).

### Identification and quantification of flavonoids

The chromatographic system for separating flavonoids was similar to that used for phenolic acids. The oven temperature was set at 34°C. The mobile phases consisted of A (0.1% acetic acid solution in water) and B (0.1% acetic acid in acetonitrile) in the following concentration gradients and flow: 1.0 mL minute^−1^, of 15% B and 85% A during the first 5 minutes; 1.5 mL minute^−1^ of B, increasing to 29%, and of A, decreasing to 71%, from 5 to 23 minutes; 1.0 mL minute^−1^, of B, increasing to 35%, and of A, decreasing to 65%, from 23 to 44 minutes; 1.0 mL minute^−1^, of B, increasing to 50%, and of A, decreasing to 50%, from 44 to 46 minutes; and 1.0 mL minute^−1^ of B, decreasing to 15%, and of A, increasing to 85%, from 46 to 48 minutes.

### Preparation of stock solutions for flavonoids

The preparation of the stock solutions consisted of dissolving 10 mg of each flavonoid in 10 mL of 80% methanol and then serially diluting the solutions in 80% methanol: 5, 10, 25 and 50 µg mL^−1^ of catechin; 25, 50, 100 and 250 µg mL^−1^ of quercetin; 2.5, 5, 10, 25 and 50 µg mL^−1^ of kaempferol-3-glucoside; 10, 25, 50 and 100 µg mL^−1^ of kaempferol-3-O-rutinoside; 10, 25, 50 and 100 µg mL^−1^ of kaempferol; and 10, 25, 50 and 100 µg mL^−1^ of quercetin-3-glucoside. The flavonoid content was expressed as microgram per gram of extract (µg g^−1^).

### Statistical analysis

Results were expressed as the mean of 3 independent biological experiments and each biological experiment was done in duplicate. Variance analysis between groups was performed using F test followed by a Tukey post hoc test. A p-value of ≤0.05 was deemed as statistically significant using *Software Statistical Analysis System* [17].

## Results and Discussion

### Determination of antioxidant capacity using radical DPPH

**Figure 1:**
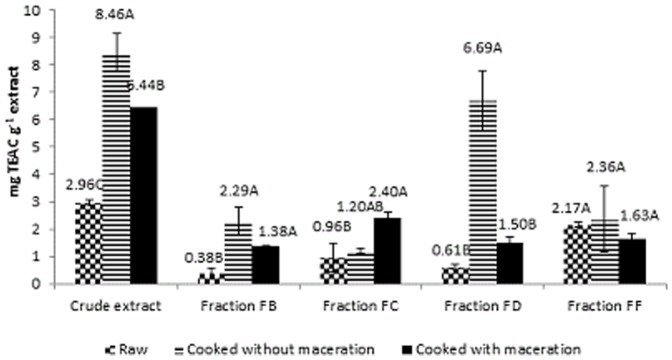
Antioxidant activity (mg TEAC g^−1^ extract) measured, using DPPH, of crude extracts and fractions of polyphenols in raw, cooked, cooked and macerated white beans.

Based on the DPPH method, the crude extract of cooked beans, with or without maceration (8.46 mg TEAC g^−1^ extract and 6.44 mg TEAC g^−1^ extract, respectively), had a higher antioxidant activity than the raw beans (2.96 mg TEAC g^−1^ extract) (Figure 1). These results are similar to those reported by Rocha-Guzman et al. [6], who concluded that the baked beans had a higher capacity of removing free radicals compared to the raw beans, though the colour of the beans and the content of phenolic compounds were reduced. This increase in the potential antioxidant activity after heat treatment, with or without maceration, may be due to the concentration of phenolic compounds in the cooking broth, which facilitated their extraction compared to the raw seeds [18].

With respect to the fractions, the cooked beans, with or without maceration, had a higher antioxidant activity compared to the fractions of raw beans due to the facility of extraction from cooked beans (Figure 1). This increased antioxidant activity from the maceration did not agree with the results obtained by Ranilla et al. [19].

Using the DPPH method, Chang and Xu [20] found that the free radical scavenging capacity was reduced by 28 to 36% in pressure-boiled beans compared to that of the raw beans and reduced by 23–31% compared to the beans that had been macerated prior to cooking. The authors attributed this reduction in the antioxidant activity to the solubilisation of soluble antioxidant compounds in the discarded soaking water and to a temperature effect.

The antioxidant activities obtained in this study (Figure 1) varied greatly among the fractions and were lower than those obtained from the crude extract. The results suggest that there might be a synergistic mediated antioxidant activity of the phenolic compounds when they are all present in the crude extract; which is not evident when these compounds are present in isolated fractions.

### Determination of antioxidant capacity using radical ABTS

**Figure 2:**
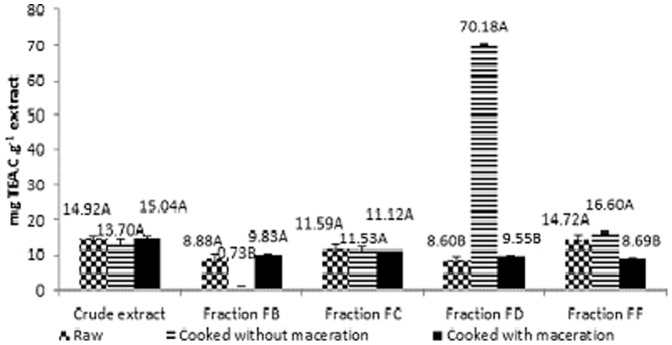
Antioxidant activity (mg TEAC g^−1^ extract) measured, using ABTS of crude extracts and fractions of polyphenols in raw, cooked, cooked and macerated white beans.

The antioxidant activity results of raw and cooked beans, with or without maceration (14.92 mg TEAC g^−1^ extract, 15.04 mg TEAC g^−1^ extract and 13.70 mg TEAC g^−1^ extract, respectively) (Figure 2) from the ABTS differed from those obtained by Madhujith and Shahidi [21], who evaluated the antioxidant activity using ABTS in acetone extracts of bark and whole beans from four types of beans. The authors obtained higher results for the bark extracts (40.74 to 46.69 times more effective than Trolox) than for the whole beans extracts (4.64 to 8.84 times more effective than Trolox). They concluded that the antioxidant activity of the extracts was higher for the red beans, followed by the brown, black and white beans. This study used a different extraction method, which might explain the difference in the obtained antioxidant capacity.

For the crude extract, the antioxidant activity was intermediary when compared with bean fractions. In addition, no significant difference was observed between the raw and cooked forms, with or without maceration, for most of the extracts, the values had variance from 0.78 mg TEAC g^−1^ extract to 70.18 mg TEAC g^−1^ extract (Figure 2). Both the ABTS and DPPH methods indicated the ability of the antioxidants to donate hydrogen atoms [21], [22]; however, heat processing reduced the sensitivity of the ABTS method in comparison to other methods (e.g., DPPH, ferric reducing antioxidant power - FRAP and oxygen radical absorbance capacity - ORAC) [22].

The reduced antioxidant activity of some fractions analysed (Figure 2), after the thermal treatment, can be explained in part by possible chemical changes, the decomposition of phenolic compounds or by the formation of complexes between proteins and polyphenols [20].

Overall, the antioxidant activity of crude extracts was comparable to their respective fractions, and the same finding was obtained by Beninger and Hosfield [5]. These findings suggest that the flavonoids present in the crude extract were further concentrated in the fractions that were analysed for antioxidant activity (Figure 1 and Figure 2).

### Identification and quantification of phenolic acids

**Figure 3:**
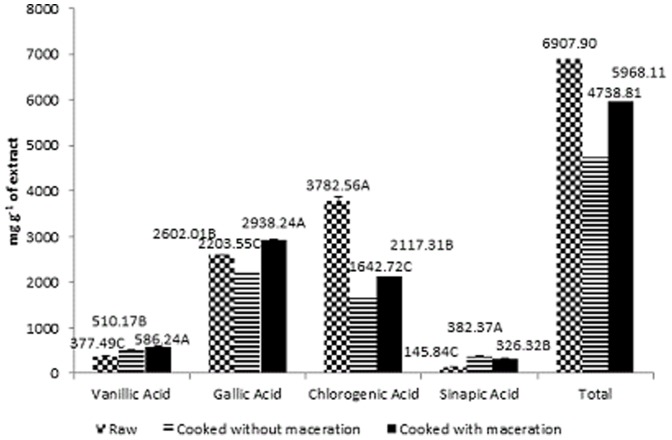
Phenolic acids (mg g^−1^ of extract) in the crude extracts from raw, cooked, and cooked and macerated white beans.

For the crude extracts all treatments contained vanillic acid, gallic acid, chlorogenic acid, sinapic acid (Figure 3). Gallic acid and chlorogenic acid were the most predominant throughout the treatments. Similar quantities of these phenolic acids have been reported in the literature [19], [23]. Phenolic acids may play important roles on the antioxidant activities, and chlorogenic acid and gallic acid give contribution to obtain the overall result. The content of phenolic compounds varies according to the cultivar and the growing conditions. Luthria and Pastor-Corrales [24] evaluated 15 types of beans and detected only p-coumaric, ferulic and sinapic acids; while the latter was found in the highest concentration. Furthermore, Ranilla et al. [25] detected chlorogenic acid only in three of the twenty-eight analysed cultivars. However, it is noteworthy that genotype, agricultural practices, climatic conditions and maturity at harvest affect the phenolic profile in this legume [18].

Cooking after maceration significantly increased the concentration of vanillic acid (586,24 mg g^−1^ of extract), cooked without maceration (510,17 mg g^−1^ of extract) and raw (377,49 mg g^−1^ of extract) presented the lower concentration (Figure 3). Maybe extraction of vanillic acid was increased by heat treatment with maceration. Contrary to this result, Diaz-Batalla et al. [26], after analysing 14 beans, found lower levels of vanillic acid in the boiled beans compared to the raw beans. Aguilera et al. [27] found no vanillic acid in baked beans, with or without maceration, and reported 10.71 mg g^−1^ in raw beans. This phenolic acid, vanillic acid, is of interest because of its anthelmintic and antisickling activities [28]. Additionally, this phenolic acid acts as a suppressor of liver fibrosis during chronic liver diseases [28], [29].

For gallic acid (Figure 3), the highest concentrations were found in the extracts of cooked beans with maceration (2,938.24 mg g^−1^ of extract). Recent studies have reported on the antiangiogenic, antioxidant, bacteriostatic and antineoplastic effects of this acid [29]. Gallic acid might be useful in the treatment of brain tumours and prostate cancer [30]. It also inhibits the activity of disaccharidases in the mammalian intestinal brush border and induces apoptosis and/or necrosis of cancer cells [29], [30]. Because of this broad range of activities, consumption of beans is beneficial to the body. Gallic acid was found in high concentrations in the fractions of beans cooked with maceration. Fraction B present 3,688.13 mg g^−1^ of extract and fraction C 2,829.65 mg g^−1^ of extract (Table 1).

**Table 1:** Phenolic acids (mg g^−1^ extract) fractions of polyphenols in raw, cooked, and cooked and macerated white beans.

	Raw	Cooked without maceration	Cooked with maceration
Vanillic Acid			
FB	-	-	677.76±0.24A
FC	83.33±0.47^a^A^b^	238.88±5.03A	442.83±1.85A
FF	41.48±26.57B	30.75±4.60A	-
Total	124.81	269.63	1120.59
Gallic Acid			
FB	-	-	3688.13±27.55A
FC	916.61±1.98B	995.59±11.48	2829.65±1.20A
FF	1881.15±245.80A	Nd	-
Total	2797.76	995.59	6517.78
Chlorogenic Acid			
FB	-	-	1398.16±1.46A
FC	1155.27±1.50B	719.85±1.20A	1366.44±1.60A
FF	1620.58±47.18A	72.05±0.86A	-
Total	2775.85	791.9	2764.6
Sinapic Acid			
FB	-	-	500.72±5.00A
FC	78.67±0.18A	149.53±0.55A	310.24±0.24A
FF	53.54±2.11A	47.48±0.07A	-
Total	132.21	197.01	810.96

a Average of two replicates ± standard deviation.

b Means with uppercase letters within a column are significantly different (≤0.05) according to the average test FB, FC and FF: fraction of polyphenols obtained using open column vacuum chromatography that had higher antioxidant activities. Two fractions for each treatment were used. Nd - not detected.

The chlorogenic acid was present in high quantities in the raw bean crude extract and fractions. Raw crude extract presented 3,782.56 mg g^−1^ of extract, fraction C 1,155.27 mg g^−1^ of extract and fraction F 1,620.58 mg g^−1^ of extract (Figure 3 and Table 1). The effect of chlorogenic acid in preventing Alzheimer's disease might be attributed to its ability to reduce apoptosis induced by the amyloid beta-cells [30], [31]. Additionally, chlorogenic acid displays anticholinesterase, antiamnesic, anti-inflammatory and antioxidant activities [30]–[34]. Chlorogenic acid increases plasma homocysteine levels, which constitutes a risk factor for the onset of cardiovascular diseases. This phenolic compound can be easily oxidised by polyphenol oxidases, leading to interactions with the NH_2_ groups of amino acids and resulting in a reduction of the nutritional value of foods. Chlorogenic acid reduced with the cooking process (Figure 3 and Table 1) [30], [35].

The highest levels of sinapic acid were found in the cooked bean crude extract without maceration (382.37 mg g^−1^ of extract). The boiled and macerated crude extract had the second highest sinapic acid levels (326.32 mg g^−1^ of extract), and the raw bean crude extract had the lowest sinapic acid values (145.84 mg g^−1^ of extract) (Figure 3). Espinosa-Alonso et al. [36] found that among four phenolic acids in beans, sinapic acid was present in the highest concentrations. Campos-Vega et al. [37] reported that the amount of sinapic acid in common beans followed that of ferulic acid. The authors reported that the phenolic acids have beneficial effects on human health due to their antioxidant activity.

Table 1 shows the results of the phenolic acids fractions present in the crude extracts, total amount ranged from 124.81 mg g^−1^ extract to 6,517.78 mg g^−1^ extract. We analysed the two fractions that had the highest antioxidant activity using DPPH and ABTS, and these fractions varied according to the treatment.

Gallic acid was not present in one of the evaluated fraction F of cooked beans without maceration (Table 1). Thus, the significant antioxidant activity present in the fractions is not due to specific phenolic compounds, which are present in crude extracts.

The highest concentrations of phenolic acids in fractions were obtained for gallic acid (995.59 mg g^−1^ extract to 6,517.78 mg g^−1^ extract) and chlorogenic acid (791.90 mg g^−1^ extract to 2,775.85 mg g^−1^ extract) (Table 1). Among the fractions evaluated for the different phenolic compounds, FB and FC had higher values than FF, although many values did not differ significantly as polarity of the acids and solvents used in the fractionation process. Phenolic acids are generally polar [38].

According to the literature, chlorogenic acid, gallic acid and sinapic acid have a high antioxidant activity while activity of vanillate is more moderate [38]–[40].Thus, bean extract fractions, which contained higher amounts of chlorogenic acid and gallic acid, have the potential to act as antioxidants, confirming the results of the antioxidant activity measured using the DPPH and ABTS methods (Figure 1, Figure 2 and Table 1).

### Identification and quantification of flavonoids

**Figure 4:**
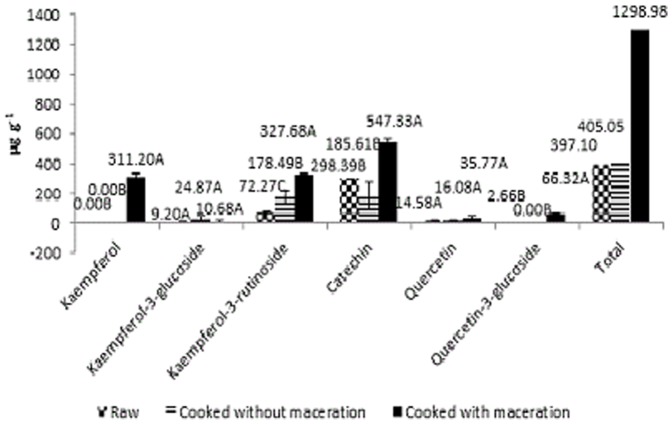
Flavonoids (µg g^−1^) of crude extracts of polyphenols from raw, cooked, and cooked and macerated white beans.

Most flavonoids were detected in almost all of the treatments, except for kaempferol, which was only detected in the cooked and macerated samples (311.20 µg g^−1^ extrat) (Figure 4). However, in a study by Diaz-Batalla et al. [26], the concentration of kaempferol was higher in the raw beans (average of 52.3 µg g^−1^) than in the cooked beans (average of 27.2 µg g^−1^ of beans). Similar results were obtained by Amarowicz and Pegg [3]. The cooking process reduced the kaempferol content by 5–71%. When assessing the levels of some flavonoids in Italian beans, Romani et al. [41] obtained trace amounts of quercetin-3-glucoside, which in this study was only found in the extract of baked beans without soaking. Nevertheless, the distribution of these compounds in the methanol extracts was highly variable depending on the applied treatments (Figure 4).

The flavonoids found in the highest concentrations were catechin and kaempferol-3-rutinoside (Figure 4). Light pigmented beans were directly correlated to a lower concentration of glycosylated flavonoids. Ranilla et al. [25] detected kaempferol in white beans; Lin et al. [42] detected myricetin-3-glucoside, quercetin-3-glucoside and kaempferol-3-glucoside in black beans and kaempferol-3-glucoside in brown beans.

The high concentration of catechin was expected as it is a type of procyanidin, which is the main group of flavonoid present in beans with light colour [43].

There was a significant difference in the flavonoid levels among the raw extracts and the cooked extracts; the crude extracts had lower flavonoid value (397.1 µg g^−1^ extract) (Figure 4). These results are contrary to previous studies that reported that cooking and steeping have negative effects on the concentrations of flavonoids [16], [26], [27].

It is noteworthy that quercetin and kaempferol-3-glucoside were not affected by cooking, either with or without maceration (Figure 4). Amarowicz and Pegg [3], however, found a 12 to 65% reduction in the quercetin concentrations after cooking beans. Diaz-Batalla et al. [26] obtained an average of 10.9 mg g^−1^ of quercetin in raw beans and of 6.5 mg g^−1^ of quercetin in cooked beans.

The flavonoid levels in the fractions are shown in Table 2. Some of the phenolic compounds were not detected in certain fractions. Kaempferol levels, which were similar among the crude extracts (Figure 4), were only present in the cooked and macerate beans fractions.

**Table 2:** Flavonoids (µg. g^−1^ extract) fractions of polyphenols from raw, cooked, and cooked and macerated beans.

	Raw	Cooked without maceration	Cooked with maceration
Kaempferol			
FB	-	-	561.20±0.48^a^A^b^
FC	0.00	0.00	304.64±21.20B
FF	0.00	0.00	-
Total	-	-	865.84
Kaempferol-3-glucoside			
FB	-	-	16.08±1.65A
FC	Nd	Nd	37.60±2.23A
FF	Nd	5.31±0.27	-
Total		5.31	53.68
Kaempferol-3-rutinoside			
FB	-	-	Nd
FC	7.23±0.47A	116.08±1.65A	Nd
FF	15.86±1.43A	30.78±0.25A	-
Total	23.09	146.86	-
Catechin			
FB	-	-	657.39±7.06A
FC	125.52±0.25A	46.17±1.60A	87.49±0.66A
FF	44.02±7.06B	7.90±1.98A	-
Total	169.54	54.07	744.88
Quercetin			
FB	-	-	67.28±0.75A
FC	Nd	16.35±9.66	29.23±2.37A
FF	9.16±0.32	Nd	-
Total	9.16	16.35	96.51
Quercetin-3-glucoside			
FB	-	-	Nd
FC	Nd	Nd	Nd
FF	6.13±0.20	Nd	-
Total	6.13	-	-

a Average of two replicates ± standard deviation.

b Means with uppercase letters within the same column are significantly different (p≤0.05) according to the test medium. FB, FC and FF: fraction of polyphenols obtained by open column vacuum chromatography that had higher antioxidant activities. Two fractions for each treatment were used. Nd - not detected.

Catechin was the only flavonoid found in all of the analysed fractions; this could be explained by the fact that this procyanidin compound belongs to the group that is present in all of the beans (Table 2). Aguilera et al. [27] reported catechin concentrations in raw and cooked white beans with maceration of 142.58 µg.g^−1^ and 76.25 µg.g^−1^, respectively; these results were very similar to those obtained in this study (Table 2).

Almost all of the analysed flavonoids had lower concentrations in the FF fraction than in the other fractions of the same treatment (Table 2). This observation leads to the conclusion that most of these phenolic compounds are either not extracted or are extracted in small quantities by water and methanol.

Though Arabbi et al. [44], Beninger and Hosfield [5] and Lin and Tang [45] reported that flavonoids occur mainly in the glycosylated form; fractions also had flavonoids in the aglycone form.

The presence of almost all flavonoids in significant quantities, especially after cooking, corresponds to how the beans are consumed in the diet. This further, confirms the importance of incorporating this food into a diet to become a diet rich in bioactive compounds. All of the analysed flavonoids (Table 2) have free radical scavenging activity. Additionally, kaempferol reduces the risk of cancer development. Other flavonoid, kaempferol-3-glucoside, has neuroprotective and anticancer effects, helps in the prevention of Parkinson's and Alzheimer's diseases. Catequina assists in reducing the intestinal absorption of cholesterol and inhibits the oxidation of LDL cholesterol. Quercetin has various effects in the prevention and treatment of some types of cancer, the reversal of cognitive disorders and the inhibition of the formation of H_2_O_2_ and histamine [4], [30], [37], [41].

In general, the heat treatment increased the antioxidant activity and the concentration of the evaluated phenolic compounds. This increase is important because raw beans has antinutritional factors and should therefore be cooked. The maceration, conversely, had variable effects on each of the analyses. Generally, phenolic acids and flavonoids increased with maceration (Table 1, Table 2, Figure 3 and Figure 4). For the antioxidant activity by DPPH demonstrated high value only for fraction B and C and by ABTS high only for fraction B.

Because of the benefits of flavonoids and phenolic acids the white beans must be included in the diet.
